# Investigating the role of transgenerational trauma in the pathogenesis of dementia: Mechanisms and long‐term impact

**DOI:** 10.1002/alz70855_102544

**Published:** 2025-12-23

**Authors:** Sarah Bauermeister

**Affiliations:** ^1^ Dementias Platform UK ‐ University of Oxford, Oxford, United Kingdom; University of Oxford, Oxford, Oxfordshire, United Kingdom

## Abstract

**Background:**

Adversity and trauma are postulated to account for the rise of multiple psychiatric disorders long after the events have taken place. This is thought to be due to the permanent changes that occur in the nervous system due to oxidative stress arising from the event. A proposed mechanism this occurs is through trauma born epigenetic imprints and defects on the Hypothalamic‐Pituitary‐Adrenal Axis which in turn affect the lateral habenula structure. These changes and resultant effects produce an environment which increases the risk for a population to gain dementia. We hypothesise that due to the epigenetic nature of generational trauma transmission, the greater associated risk of acquiring dementia would be passed down to future generations.

**Methods:**

We would use murine models to experimentally look at how trauma infliction affects the neuroanatomy of prenatal mice and explore how biologically and socially their offspring behaves because of their parents’ history (with our collaborators at the Korean Brain Research Institute). This would be through analysis of lateral habenula structure and methylation assay analysis compared to control murine samples. We would compare the finding with the human longitudinal cohorts who have suffered from events of extreme adversity and see how dementia frequency and outcomes are exacerbated compared to normal populations (which would be completed by creation of a composite score from available risk‐related variables).

**Results:**

Analysis of these datasets would help to corroborate the link between trauma and its ability to disrupt the normal development of the nervous system. It would also help establish whether those descending from parents who faced extreme adversity are more at risk to develop dementia causing conditions. The associations between the murine and human models would be realised by translating findings through common variables.

**Conclusion:**

The overarching outcome would be to investigate through what means and the extent to which generations that hail from backgrounds of trauma and adversity are more likely to develop dementia. This work will provide a rich source of information in generational studies of dementia and also make people affected by these conditions to be more proactive with reducing other dementia risk factors.

